# Current and Emerging Pharmacological Treatment Options for Dementia

**DOI:** 10.1155/2006/315386

**Published:** 2006-05-11

**Authors:** John M. Ringman, Jeffrey L. Cummings

**Affiliations:** Department of NeurologyAlzheimer's Disease Research Center; University of CaliforniaLos AngelesCAUSA

**Keywords:** Dementia, treatment, Alzheimer’s disease, vascular dementia, frontotemporal dementia, dementia with Lewy Bodies, Parkinson’s disease, acetylcholinesterase inhibitors, memantine, review

## Abstract

Treatments for the symptomatic relief of Alzheimer’s disease are available but despite advances in our ability to treat persons with various forms of dementia, more effective treatments are needed. The cholinesterase inhibitors donepezil, rivastigmine, and galantamine have demonstrated efficacy in improving cognition and global status and to a lesser extent, behavioral abnormalities relative to placebo in patients with mild-to-moderate Alzheimer’s disease. Rivastigmine has been shown to benefit patients with dementia with Lewy Bodies and with dementia associated with Parkinson's disease. Donepezil and galantamine have also been shown to be mildly effective in dementia due to cerebral ischemia. Memantine has a distinct mechanism of action and is effective in moderate-to-severe AD. The benefits from these drugs, however, are limited and their long-term effectiveness has not been well-demonstrated. Their clinical utility is controversial. Many novel approaches that promise to provide more effective treatments are currently being pursued.

## 1. Introduction

Between the years 1900 and 2000, life expectancy at birth in the US increased from 47 to 77 years (www.cdc.gov/nchs/datawh.htm) and for many individuals health and productivity extend into these additional years. Unfortunately, the prevalence of dementia increases geometrically after the age of 65 years [31], resulting in diminished quality of life for affected persons as well as their loved ones and an increased financial burden to individuals and society. The development of efficacious interventions to prevent, slow the progression of, diminish the symptoms of, or even reverse the pathology of dementing conditions is therefore a priority. Though we have a long way to go, some advances have been made and are the topics of this review. In most autopsy series, Alzheimer's disease (AD) is the most common form of dementia, accounting for approximately two-thirds of cases [34]. Alternative pathological processes that contribute to dementia, however, are not uncommon and may be the sole cause of dementia or co-exist with AD or each other to induce cognitive deficits [42]. Other neurodegenerative processes such as those associated with the intracellular formation of Lewy bodies and those underlying the clinical phenotypes of frontotemporal dementia are not uncommon. Cerebrovascular ischemia can cause dementia as well and its contribution to the clinical status of a specific patient may be difficult to estimate. Because of its prevalence, treatment of AD has been emphasized over that of other dementing conditions in drug development. Importantly, however, there have been efforts to develop medications for and test therapies in these other dementing illnesses as well. We will review these.

ISSN 0953-4180/06/$17.00 © 2006 – IOS Press and the authors. All rights reserved

## 2. Alzheimer's disease

AD has many pathological manifestations including extracellular amyloid plaque deposition, intracellular neurofibrillary tangle formation, and ultimately neuronal and synaptic loss with consequent impairment of neurotransmission [13]. Treatments targeting all of these processes have either been developed or are currently under investigation.

### *2.1. Acetylcholinergic neurotransmission*

Though the neuronal loss occurring in AD is widespread and ultimately involves many cell groups, early neurochemical studies of the brains of persons dying with AD revealed a relatively greater extent of loss of neurons employing acetylcholine as a neurotransmitter [14,71]. As these networks were known to be involved in memory and attention, aspects of cognition that are affected in AD, chemically enhancing the function of remaining cholinergic neurons became a focus of treatment development. Attempts were made to enhance cholinergic neurotransmission by increasing the availability of precursors for acetylcholine [49] and by oral and intraventricular administration of direct muscarinic receptor agonists [53]. Progress was eventually made with medications that inhibit the enzyme that degrades acetylcholine (acetylcholinesterase or simply cholinesterase) and four of these have now received Food and Drug Administration (FDA) approval in the US. Despite their limited efficacy, they have become the cornerstone of AD treatment.

### *2.2. Tacrine*

Tacrine was the first medication to be approved by the US FDA for the treatment of AD. It was demonstrated to be effective in improving cognition and global status compared to placebo in persons with mild-to-moderate probable AD in a 12-week randomized double-blind, placebo-controlled trial [22]. Now rarely used secondary to a high incidence of hepatoxicity and its difficult dosing regimen, the development of tacrine helped to establish the paradigm by which potential drugs for AD are evaluated.

### *2.3. Donepezil*

Of the currently available cholinesterase inhibitors, clinical and research experience with donepezil is the most extensive and therefore it has become a standard with regard to considering the efficacy of the cholinesterase inhibitors as a class. Donepezil is a reversible cholinesterase inhibitor that was shown in two independent randomized studies to be superior to placebo after 12 and 24 weeks at 5 and 10 milligram (mg) per day in delaying apparent decline of cognitive function on the Alzheimer's Disease Assessment Scale – cognitive subscale (ADAS-Cog) in persons with mild-to-moderate AD [55,56]. The difference in mean scores between donepezil- and placebo-treated patients was approximately three points on this 70-point scale. The difference between donepezil- and placebo-treated patients was also evident on the Clinician's Interview Based Impression of Change plus caregiver input (CIBIC+), a global scale of clinical status based on a structured interview with the patient and a caregiver. In both of these studies discontinuation of donepezil resulted in a decline on the ADAS-Cog. This decline was more dramatic in the 24 week study in which subjects were re-assessed after a 6 week washout than in the 12 week study in which subjects were re-assessed at 3 weeks. Both studies had extension phases in which all patients received open-label donepezil after the washout period [16]. The group that had formerly been on donepezil did not demonstrate a differential response from those who had been on placebo in the 24 week study with a 6-week washout whereas those who were formerly treated with donepezil continued to do better than those treated previously treated with placebo in the 12 week study with a 3-week washout. This suggests that the effect of donepezil is more thoroughly negated after 6 than after 3 weeks of abstinence.

A one-year, placebo-controlled study of donepezil in AD was performed in Europe that employed different scales as end-points and provided further evidence that treatment with donepezil had benefits in overall course as well as for cognition and activities of daily living (ADLs) over this time interval [73]. Another one-year placebo-controlled study in the US demonstrated that treatment with donepezil significantly decreased the likelihood of progression of functional decline during this interval [44]. Longer placebo-controlled studies are now ethically difficult to do secondary to the demonstrated efficacy, though small in degree, of cholinesterase inhibitors.

In a study sponsored by the manufacturers of donepezil, patients previously enrolled in placebo-controlled studies and then given open-label donepezil were followed longitudinally. Time to nursing home placement was compared between those who were estimated to have received an effective dose of donepezil (defined as at least 5 mg/day, 80% of the time or greater) and those who did not [28]. Compliant patients were found to have nursing home placement delayed by around 21 months. It is possible that differences between patients and their caregivers who were compliant and patients and caregivers that were not compliant account for at least part of this effect [25].

It is argued that the consistent yet small benefits of donepezil on cognitive and global assessments are not clinically significant and therefore long-term treatment with donepezil is not cost-effective. Investigators in the U.K. set out to ascertain the long-term effects of donepezil vs. placebo on nursing home placement and progression of disability in a population of persons with dementia not as strictly defined as AD as in most prior drug studies [10]. These authors also collected cognitive and neuropsychiatric measures as secondary outcomes in addition to performing an economic evaluation. They confirmed a small benefit of donepezil on Mini-Mental Status Examination (MMSE) scores and on a scale measuring activities of daily living after two years but failed to find a significant effect on institutionalization, progression of disability, or behavioral symptoms. They also concluded that treatment with donepezil was associated with higher overall costs than placebo and was therefore not cost-effective. An unusual feature of this study was periods of drug washout, particularly a six-week washout between the first and second 48-week treatment intervals. Although this study poignantly emphasized the limited benefits of donepezil, it should not be over-interpreted.

Donepezil is administered orally once per day. Treatment is initiated at 5 mg/day which can be increased to 10 mg/day with a decreased likelihood of side effects with slower titration. It is generally tolerated well with transient adverse effects occurring less than 10% of the time.

### 2.4. *Rivastigmine*

Rivastigmine is a carbamate-based cholinesterase inhibitor that binds to the esteratic site of the enzyme but dissociates slowly and its effect is therefore described as “pseudoirreversible.” Degradation by butyryl- and acetycholinesterase is the principal mechanism of metabolism of rivastigmine and it therefore has few drug-drug interactions [64].

Rivastigmine was demonstrated to have an effect superior to placebo on the ADAS-Cog and CIBIC+ as well as on the MMSE and two scales of overall disease progression after 26 weeks in persons with mild-to-moderate AD [9]. High dose (6–12 mg/day) had a greater effect than low dose (1–4 mg/day) rivastigmine on all measures with the difference between placebo and high-dose rivastigmine-treated groups on the ADAS-Cog being 5 points. The drop-out rates due to adverse effects were greater on the higher dose (29% vs. 8%) of rivastigmine. A parallel study in Europe showed similar efficacy and tolerability results [57].

Attempts have been made to ascertain the long-term effects of treatment with rivastigmine by comparing patients that received the drug chronically to those that were not treated for some period of time. In an open-label extension of the 26-week pivotal trial described above, patients initially on placebo were subsequently put on rivastigmine with the dose increased as tolerated up to a maximum of 6 mg b.i.d [21]. An initial improvement in the mean ADAS-Cog score was seen in this group (mean daily dose 8.5 mg) that after 52 weeks was statistically lower than the group treated with high-dose rivastigmine (mean dose 9.4 mg) throughout. The same authors analyzed the ADAS-Cog scores in patients from the 26-week placebo-controlled studies that were originally treated with high-dose rivastigmine for any length of time but who had subsequently dropped out for various reasons [23]. Among the subset of such patients whose 26-week ADAS-Cog scores were available (88/313), mean ADAS-Cog scores had not deteriorated as greatly as those who were treated with placebo throughout. These data have been interpreted as indicating an effect of rivastigmine on the progression of AD but the results could also be due to the selected nature of the subjects included in the analysis and/or by prolonged symptomatic effects of rivastigmine.

Rivastigmine is administered twice daily in doses from 1.5 mg/day to 6 mg. b.i.d. The adverse effects of rivastigmine are similar in nature to that of donepezil and other cholinesterase inhibitors. However, they are more frequent and severe at higher doses of rivastigmine and titration of the medication should be performed slowly with vigilance to emerging nausea and vomiting. Weight loss occurs in some patients treated with rivastigmine.

### 2.5. Galantamine

Galantamine is a tertiary alkaloid that has been isolated from various plants and is a competitive and reversible inhibitor of acetylcholinesterase [30]. It has been used in Europe by anesthesiologists to help reverse paralysis induced by curare-like muscle relaxants and was approved for use in patients with AD in Austria prior to 1995. In addition to inhibiting acetylcholinesterase, it may enhance cholinergic neurotransmission by allosterically sensitizing nicotinic receptors to the effects of acetylcholine [38].

In a prospective study of 978 patients randomized to placebo, 8, 16, or 24 mg of galantamine per day in two divided doses, the difference in mean change in ADAS-Cog scores between the placebo and 24 mg treatment groups was 3.6 in the observed case analysis after 21 weeks of treatment ($p < 0.001$) [68]. Change in CIBIC+ ($p < 0.001$) and total Neuropsychiatric Inventory (NPI) scores ($p < 0.05$) were also significantly better than placebo in the 24 mg galantamine group in this study and this group did not differ from the 18 mg/day group on these measures. Dosing changes in this study were made every 4 weeks and rates of nausea were 13% and 17% in the 16 and 24 mg/day galantamine group compared to the placebo rate of 5%. In another study, patients were randomized to receive placebo, 24 mg/day or 32 mg/day of galantamine [52]. After 6 months the 24 mg/day and 32 mg/day galantamine groups had better mean changes in ADAS-Cog/11 (a subtest of the ADAS-Cog) score and on the CIBIC+. In this study dose increases were made at one week intervals and the resulting rates of nausea were 13% for placebo, 37% for the 24 mg/day group, and 44% for the 32 mg/day galantamine group. At 6 months' time, patients on placebo were switched to 24 mg/day whereas those on 32 mg/day were switched to 24 mg/day for another 6 months. The ADAS-Cog/11 scores initially improved in subjects formerly given placebo though at 6 months' time they scored significantly lower on this scale than subjects treated with 24 mg of galantamine throughout the study.

When data from two separate two-year studies in which the utility of galantamine in persons with a memory disorder who did not otherwise meet criteria for dementia (mild cognitive impairment, or MCI) were combined, a statistically significant excess number of deaths was seen in the galantamine group (13/1026) relative to the placebo-treated group (1/1022). The number of deaths in the placebo group is less than would be expected under normal circumstances in this population and chance occurrence probably explains this finding. There is no obvious reason to suspect that persons with MCI are more susceptible to death from galantamine therapy but the galantamine package insert has nonetheless been modified to reflect this observation.

## 3. Acetylcholinesterase inhibitors and neuropsychiatric symptoms

AD is characterized not only by memory and other cognitive deficits but also by behavioral changes that are frequently the cause of institutionalization [32,41]. There is ample reason to suspect that loss of cholinergic neurons underlies not only the cognitive deficits but also contributes to such behavioral symptoms [14]. Treatment with cholinesterase inhibitors can help ameliorate these disturbances in some instances.

In double-blind randomized clinical trials in AD the NPI is a frequently used outcome measure [15]. The NPI is a structured interview in which an appropriate caregiver is questioned about twelve different categories of behavioral symptoms. In a placebo-controlled study of donepezil in mild-to-moderate AD, no differences were seen in NPI score change after 1 year between treatment groups [73]. This might reflect the low baseline level of psychopathology in subjects on entry. In a 24-week trial of donepezil in patients with more advanced AD, significant improvement was seen on the total NPI score in donepezil-treated subjects [24]. Another study of AD patients in nursing homes did not demonstrate changes in total NPI score but did find significant differences in the agitation/aggression sub-item score favoring donepezil [66].

In a 26 week placebo-controlled study of rivastigmine, patients who received 6–12 mg/day had improvement on behavioral sub-items of the CIBIC+ [58]. Aggressiveness, hallucinations, and paranoid features were particularly impacted. In the 5-month galantamine study [68], the groups treated with 16 mg and 24 mg/day of galantamine demonstrated statistically significantly better total NPI scores than did the placebo group. Among observed cases that had aberrant motor behavior, agitation/aggression, and anxiety at baseline, those treated with galantamine 16 or 24 mg/day showed significant decreases of these disturbances compared to placebo. Apathy, aggressiveness/agitation, hallucinations, and delusions are among the symptoms that have been reported to be improved with cholinesterase inhibitors.

In a meta-analysis of studies looking at the effects of cholinesterase inhibitors on neuropsychiatric symptoms, differences favoring treatment over placebo were seen both in studies employing the non-cognitive subportion of the ADAS (ADAS-Noncog) and those using the NPI [69]. Clinical experience suggests that though improvement in troublesome behaviors is sometimes seen, such a response is not always dependable.

Existing studies of the effects of cholinesterase inhibitors on neuropsychiatric disturbances in AD are limited by the fact that most have not specifically enrolled patients with such disturbances and behavioral measures were used as secondary rather than primary outcomes. The relative paucity of behavioral disturbances in subjects makes it difficult to demonstrate an improvement in these symptoms. In the future studies should be designed specifically to address this issue.

## 4. Acetylcholinesterase inhibitors – general comments

All three of the commonly used cholinesterase inhibitors have demonstrated beneficial effects on cognition and on global assessments of change compared to placebo. This has led to their widespread use in AD and to the American Academy of Neurology's (AAN) practice parameter: "Cholinesterase inhibitors should be considered in patients with mild-to-moderate AD, although studies suggest a small average degree of benefit [17]" The utility and cost-effectiveness of this small benefit has been questioned by some. In a cost-effectiveness study it was estimated that cholinesterase inhibitors would have to have a benefit sustained for at least two years in order for their benefits to outweigh their costs [45]. Unfortunately, an attempt at performing such a long-term study had many pitfalls (see above) [10] and in light of the demonstrated efficacy of cholinesterase inhibitors, another such long duration placebo-controlled study is unlikely.

The effects of these medications on cognition seem to be dose-related with donepezil and rivastigmine though this is not clearly the case with galantamine. Drop-out rates were higher in the studies of rivastigmine and galantamine. This is likely due at least in part to differences in the methods and timing of dose titration employed in the studies [3] as it has been repeatedly shown that slow titration improves tolerability.

Adverse effects of cholinesterase inhibitors are characteristic of this class of medications and include those symptoms that might be expected from cholinergic hyperactivity. Specifically, nausea, diarrhea, vomiting, muscle cramps, anorexia, and dizziness are not uncommon. There are no absolute contraindications to use of the medications but caution is advised with regards to potentially exaggerating the effect of succinylcholine-type pharmacologic paralysis, inducing bradycardia in persons with supraventricular-conduction defects, and in enhancing gastric acid secretion and therefore increasing the risk of gastrointestinal bleeding in susceptible individuals. For a comparison of major properties of the cholinesterase inhibitors, see Table 1.

### *4.1. Memantine*

There is substantial in-vitro and in-vivo evidence that excessive excitatory stimulation of neurons by the neurotransmitter glutamate on N-methyl-D-aspartate (NMDA) receptors is a mechanism of cell death involved in neurodegenerative disease [37]. Memantine is a moderate-affinity uncompetitive NMDA receptor blocker that has been used in Germany since 1982 for symptomatic treatment of Parkinson's disease and other neurological conditions. It was subsequently studied in AD and was approved by the US FDA for use in moderate-to-severe AD in late 2003.

The efficacy of memantine in a nursing home population of severely affected dementia patients (MMSE scores $<10$) was studied in Latvia [74]. In this 12-week study, in which 49% of patients were diagnosed with AD and 51% with vascular dementia, memantine was found to improve Clinical Global Impression of Change (CGI-C) scores significantly more often than placebo (73% vs. 45%) and to improve nursing staff-rated scores on the Behavioral Rating Scale for Geriatric Patients (BGP) to a greater degree. In a subsequent placebo-controlled 28 week study of memantine performed in moderate-to-severe probable AD patients (mean MMSE score $= 8$) performed in the US, memantine demonstrated superiority to placebo in the primary efficacy variables in the observed case analysis [54]. Statistical significance on the CIBIC+ was narrowly missed ($p = 0.06$) in the last observation carried forward (LOCF) analysis though scores on the Alzheimer's Disease Cooperative Study – Activities of Daily Living scale (ADCS-ADLsev) scale, the Severe Impairment Battery (SIB, a cognitive test) and the Functional Assessment Staging scale (FAST)

Table 1
A comparison of pharmacological properties of acetylcholinesterase inhibitors

| | Donepezil | Rivastigmine | Galantamine |
|---|---|---|---|
| Absorption affected by food | No | Yes | Yes |
| Serum-half life (hr) | 70–80 | 2 | 5–7 |
| Protein binding (%) | 96 | 40 | 0–20 |
| Metabolism | CYP2D6, CYP3A4 | Non-hepatic | CYP2D6, CYP3A4 |
| Maximal Dose | 10 q.d. | 6 mg b.i.d. | 12 mg b.i.d. |
| Rates of nausea in clinical trials (%) | 17% | 48% | 37% |

(OR – AS A CAPTION): Table 1. A comparison of pharmacological properties of acetylcholinesterase inhibitors used for the treatment of Alzheimer's disease.

all favored the memantine-treated group in this analysis.

Because of the widespread use of acetylcholinesterase inhibitors, it is useful to know the effects of their use in combination with memantine. A randomized, placebo-controlled 24-week study of memantine in patients with moderate-to-severe AD already on stable doses of donepezil showed that those treated with memantine did better on the SIB and the ADCS-ADLsev (primary outcome measures) as well as on the CIBIC+, the NPI, and the BGP (secondary outcome measures) in the LOCF analysis [67].

Memantine, both alone and in combination with donepezil, therefore has demonstrated efficacy in the treatment of moderate-to-severe AD. Though it would be expected that "neuroprotectant" NMDA receptor antagonists should exert their effect on disease progression over the long run, experience has been that there can be an immediate improvement in treated patients' status, bringing into question the relevant mechanism of action of the drug.

Memantine is very well tolerated with adverse events occurring with essentially equal frequency in the placebo group in these controlled trials. Clinical experience with the drug, however, has shown that reversible worsening of agitation and psychosis occurs rarely. Treatment with memantine is begun at 5 mg/day and titrated upward to 10 mg/bid though the half-life of the medication (60–80 hours) suggests that once a day dosing is possible.

## 5. Anti-oxidant medications

There is substantial evidence for a role of oxidative damage in the pathogenesis of AD and other neurodegenerative diseases [50]. In light of epidemiological, in-vitro, and in-vivo data, trials of drugs with oxygen radical-scavenging properties have been undertaken in AD. One of the largest of such studies was a two-year trial of selegiline (10 mg/day), vitamin E (2,000 i.u./day), both, or placebo in which time to progression of disease to death, institutionalization, loss of basic ADLs, or to a Clinical Dementia Rating (CDR) score of 3 were the primary endpoints [60]. When baseline MMSE was included as a covariate, all three treatment groups had a delayed time of progression to endpoint compared to placebo. There was no significant difference between groups and in part because of the ease with which vitamin E may be administered, treatment with 2,000 i.u./day of vitamin E became a common practice in the treatment of AD.

A 3-year study of vitamin E or donepezil vs. placebo in persons with MCI has recently been completed [50]. In this study the primary endpoint was time of disease progression until a diagnosis of dementia was made. Donepezil had the effect of reducing the mean time of progression to a diagnosis of AD for about 6 months but at 3 years' time there were no differences in the likelihood of being diagnosed with dementia between any of the 3 groups. Vitamin E at 2,000 i.u./day had no measurable effect on progression of disease in this study.

Recently the safety of high-doses of vitamin E has been called into question. In a meta-analysis of safety in 19 prospective clinical trials involving various doses of vitamin E, an excess mortality of 39 per 10,000 patients was seen in studies employing 400 i.u./day or more [43]. As many of the deaths in this meta-analysis were due to myocardial infarction in persons known to have risk factors for coronary artery disease, caution is indicated in recommending high doses of vitamin E in similar at-risk patients. No such excessive deaths were seen in either the study of vitamin E in AD or MCI and therefore a risk of high-dose vitamin E in such patients has not been demonstrated.

Though there are safety issues with regard to the use of high dose vitamin E that need to be addressed, the potential benefit of anti-oxidants in AD requires further study. Additional studies of vitamin E as well as other anti-oxidants (e.g. alpha-lipoic acid, coenzyme Q, vitamin C, resveretrol, curcumin) are either underway or being planned.

## 6. Anti-inflammatory medications

Inflammation is also thought to play a role, if not a causative one, in the propagation of the pathological processes leading to AD [2]. Epidemiological studies have demonstrated a relationship between prior use of non-steroidal anti-inflammatory drugs (NSAIDs) and a decreased subsequent risk of developing AD [20]. However, prospective studies of traditional NSAIDs and selective cyclooxygenase 2 (COX-2) inhibitors have not demonstrated an effect on the clinical course of persons with AD [1]. Though a role of anti-inflammatory medications in preventing or slowing AD has not been entirely ruled out, the potential adverse effects of these medications militates against their current use in AD [11].

## 7. Cholesterol-lowering medications

Studies of transgenic mouse models of AD and epidemiological studies have provided support for a role of cholesterol-lowering medications in the treatment of AD [51]. Preliminary data from small studies have indicated that treatment with medications that inhibit 3-hydroxy-3-methylglutaryl coenzyme A (HMG-CoA) reductase, a critical enzyme involved in the synthesis of cholesterol, may affect the disease process in AD [33]. Larger prospective, double-blind, placebo controlled trials of such "statin" drugs are currently underway. Lack of definitively demonstrated efficacy and potential side effects of these medications preclude recommendation of their use in AD at this time.

## 8. Anti-amyloid interventions

Though it is clear that other factors are critical to the development of AD, the available genetic evidence argues for a primary role of excessive production, deposition, and/or decreased degradation of components of beta-amyloid protein in the etiology of AD [62]. It stands to reason that interventions that interfere with these processes might slow disease progression or even reverse some of the symptoms.

A large human trial aimed at increasing the reabsorption of amyloid plaques by stimulating the immune system by vaccination had to be discontinued because of an autoimmune encephalitis that occurred in 17 out of 300 patients treated [29]. Alternative immune approaches including passive immunization through the infusion of anti-amyloid antibodies are being explored.

Drugs that inhibit the enzymes that aberrantly cleave amyloid precursor protein (gamma- and beta-secretase) are also in development. In an early phase study of a gamma-secretase inhibitor, non-significant decreases in cerebrospinal fluid concentrations of Abeta1-40 were seen, possibly indicating a mild effect on abnormal metabolism of beta-amyloid protein [63].

Another approach is to use medications that interfere with amyloid aggregation. This may be achieved by directly interacting with amyloid metabolites which is thought to be the mechanism of action of NC-531 which is currently in Phase III studies [27]. The presence of certain metal ions augment the aggregation of amyloid into plaques. Drugs that bind to and remove metal ions are being studied for their efficacy in AD. One such drug, the antibiotic clioquinol, has demonstrated efficacy in animal models of AD [8] and its safety is being re-evaluated in humans.

Though promising, these approaches have been criticized as being too limited. That is, the role of other neurochemical processes (e.g., the phosphorylation of tau and consequent formation of neurofibrillary tangles) are also critical such that even if one is completely successful at reversing amyloid plaques, sufficient pathology will remain that symptoms may still be present.

## 9. Dementia associated with Parkinsonism

Dementia occurs concurrently with the motor symptoms of Parkinsonism in a number of different conditions. It can occur in association with dementia with Lewy Bodies (DLB), is seen with increasing frequency with progression of idiopathic Parkinson's disease (PD), occurs in other neurodegenerative diseases associated with parkinsonism (e.g. corticobasal ganglionic degeneration and other "Parkinson's Plus" syndromes), and it frequently exists in the presence of Parkinsonian gait abnormalities in vascular dementia (VaD). We will focus here on the treatment of DLB and of the dementia associated with PD. The management of VaD will be discussed below.

### *9.1. Dementia with lewy bodies*

Lewy bodies, intracellular eosinophilic inclusion bodies that stain positive for ubiquitin and alphasynuclein, are found in the cerebral cortex in at least 26% of patients with dementia [4]. When present, they are typically associated with the motor features of Parkinsonism, visual hallucinations, and fluctuating mental status in addition to depression, apathy, and REM behavior disorder [7,40]. Neuropathological studies of persons with DLB have demonstrated a more severe loss of cortical cholinergic input indexed by decreased levels of choline acetyltransferase with relatively preserved post-synaptic muscarinic receptors [48]. Such pathology may underlie the more dramatic psychopathology occurring in DLB and suggests the possibility of an enhanced response to cholinergic treatments.

The efficacy of rivastigmine in patients with clinically defined DLB was investigated in a multinational, randomized, double-blind, placebo-controlled study in which behavioral abnormalities as measured with the NPI and cognition measured with a computerized battery were the primary outcome measures [39]. After 20 weeks, patients treated with rivastigmine at a maximum mean dose of 9.4 mg had statistically better scores on the NPI, particularly with regard to apathy, anxiety, delusions, hallucinations, and aberrant motor behavior. The rivastigmine-treated subjects also performed better on the cognitive assessments but change in the CGIC+ was not significantly different between groups. Rivastigmine was well-tolerated without any significant worsening in Parkinsonian symptoms.

There is little reason to suspect that symptoms of DLB will respond selectively to rivastigmine as opposed to other acetylcholinesterase inhibitors but there are few data on the treatment of DLB with other cholinesterase inhibitors [5]. Amelioration of behavioral symptoms in DLB by cholinesterase inhibitors is particularly encouraging considering the sensitivity of DLB patients to dramatic motor worsening precipitated by neuroleptic medications [40].

## 10. Parkinson's disease

Approximately 40% of patients with idiopathic PD have cognitive impairment to a degree qualifying them as demented [12]. The incidence of dementia increases with the duration of PD. There is uncertainty regarding the pathological changes most closely correlated with cognitive decline in PD but a cholinergic deficit has been demonstrated in idiopathic PD [47].

A 24-week randomized, placebo-controlled study of rivastigmine in patients with PD was performed using the ADAS-Cog and CIBIC+ as primary outcome measures [18]. Five-hundred forty-one subjects were randomized of which 410 completed the study. Mean dose of rivastigmine was 8.3 mg in the 263 active treatment patients that completed the study. In the LOCF analysis, persons treated with rivastigmine had significantly superior scores on the ADAS-Cog and CIBIC+ as well as on the secondary outcome measures of the ADCS-ADL, NPI, and the cognitive tests (MMSE, computerized battery, verbal fluency, and clock drawing). Adverse effects paralleled those seen in other studies of rivastigmine though a higher rate of worsened tremor occurred in treated patients (10.2% vs. 3.9%). The results of this study suggest that rivastigmine and perhaps other cholinesterase inhibitors have benefits in cognition in idiopathic PD though may in some cases worsen tremor.

## 11. Frontotemporal dementia

Frontotemporal dementia, or FTD, is a group of neurodegenerative diseases that present with behavioral changes including apathy, depression, anxiety, compulsions and disinhibition. The cognitive changes consist of executive dysfunction and language deficits with early sparing of memory and visuospatial function relative to AD. FTD tends to present at a younger age than AD and some patients have concurrent motor neuron disease and/or parkinsonism. FTD accounts for 8–17% of dementia cases younger than 70 years of age [4,6] and is associated with at least 3 distinct pathological entities [59]. FTD is associated with deficits in multiple different neurotransmitter systems.

Controlled, randomized treatment trials in FTD are few. Clinical experience suggests that FTD does not respond to cholinesterase inhibitors. Behavioral symptoms are generally treated empirically. In a small, open-label series of 11 patients behavioral symptoms responded to serotonin reuptake inhibitors in about half [65]. Because the underlying pathology frequently demonstrates abnormal depositions of tau protein, trials of drugs that inhibit the phosphorylation of this protein are being considered. A study of memantine in FTD is currently underway.

## 12. Vascular dementia

Prevalence rates of vascular dementia (VaD) vary widely, at least in part because of inconsistencies in the definition of the condition. Cognitive impairment associated with stroke can be due to ischemic brain damage itself but it is frequently difficult to rule out the simultaneous presence of AD pathology. Furthermore, ischemic brain damage itself takes many forms including that due to cortical strokes, multiple subcortical lacunar strokes, and diffuse white matter ischemia not always associated with clinical events. Attempts to study treatments in VaD are complicated by the heterogeneity of this population. There have been many relatively small and likely underpowered studies of a variety interventions in VaD [46] but none of these have convincingly demonstrated an effect in VaD such that the American Academy of Neurology concluded in its 2001 practice parameter, "There are no adequately controlled trials demonstrating pharmacologic efficacy for any agent in ischemic vascular (multi-infarct) dementia [35]."

The recent demonstration of cholinergic deficits in patients with ischemic brain damage has inspired attempts to treat the condition with cholinomimetic drugs. The hippocampus, in which acetylcholine neurotransmission is important and which plays a critical role in memory formation, is sensitive to ischemia and is atrophied in VaD [70]. Furthermore, the cholinergic projections to cortex from the basal nucleus of Meynert pass through white matter areas known to be affected by subcortical ischemia [61].

In a 24-week study of donepezil 5 mg/day vs. donepezil 10 mg/day vs. placebo involving 616 subjects with probable (76%) or possible (24%) VaD, treatment with either dose of donepezil was superior to placebo on the primary outcome measures, the ADAS-Cog and the CIBIC-plus [72]. As the placebo-treated group did not decline on these measures, the difference was accounted for by an improvement of about 2 points on the ADAS-Cog (in the donepezil 10 mg/day group). Statistically significant improvement was also seen on the MMSE (approximately 1 point) and the CDR. There were no major differences in efficacy between the donepezil 5 mg and 10 mg group with more adverse effects in the 10 mg group suggesting that the lower dose of donepezil should be considered in patients with VaD. A large study of galantamine in VaD yielded similar results [19]. These studies provide evidence for efficacy of cholinesterase inhibitors in VaD though perhaps of a smaller degree than is seen in AD. Whether the response to cholinesterase inhibitors is due a deficit from ischemic damage or to concurrent AD pathology is currently an unresolved issue.

Ginkgo biloba is a plant extract used in Chinese herbal medicine for a variety of ailments. It is approved in Germany for the treatment of dementia. Its mechanism of action is unknown though it has anti-oxidant and platelet-inhibiting properties. In a one-year study of 309 persons with mild-to-moderate dementia (76% with AD, 24% characterized as having multi-infarct dementia), small but statistically significant differences favoring ginkgo biloba were seen on the ADAS-Cog (1.5 points) and the Geriatric Evaluation by Relative's Rating Instrument (GERRI) but not on the CGIC [36]. The effect of ginkgo biloba is small and its use in AD is not widely recommended. In part due to its action on platelet aggregation, its utility in VaD is currently being evaluated in a large study.

## 13. Conclusions

We have made progress in the pharmacological treatment of dementia in the last 15 years, but the currently available treatments have limitations. The cholinesterase inhibitors donepezil, rivastigmine, and galantamine have demonstrated efficacy in improving cognition and global status and to a lesser extent, behavioral abnormalities relative to placebo in patients with mild-to-moderate Alzheimer's disease. Rivastigmine has been proven to benefit patients with dementia with Lewy Bodies and with dementia associated with Parkinson's disease. Donepezil and galantamine have also been shown to be mildly effective in dementia due to cerebral ischemia. Memantine has a distinct mechanism of action and also has been shown to be effective in moderate-to-severe AD. There are currently no proven treatments directed specifically at the pathology of frontotemporal dementia.

The benefits of available medications for dementia are limited. Interventions that truly modify the disease process are needed. There are many promising avenues currently being pursued towards this end.
